# The Posterior Parietal Cortex Subserves Precise Motor Timing in Professional Drummers

**DOI:** 10.3389/fnhum.2017.00183

**Published:** 2017-04-11

**Authors:** Bettina Pollok, Katharina Stephan, Ariane Keitel, Vanessa Krause, Nora K. Schaal

**Affiliations:** ^1^Medical Faculty, Institute of Clinical Neuroscience and Medical Psychology, Heinrich-Heine University DuesseldorfDuesseldorf, Germany; ^2^Department of Experimental Psychology, Heinrich-Heine University DuesseldorfDuesseldorf, Germany

**Keywords:** anticipation, brain plasticity, musicians, synchronization, transcranial direct current stimulation (tDCS)

## Abstract

The synchronization task is a well-established paradigm for the investigation of motor timing with respect to an external pacing signal. It requires subjects to synchronize their finger taps in synchrony with a regular metronome. A specific significance of the posterior parietal cortex (PPC) for superior synchronization in professional drummers has been suggested. In non-musicians, modulation of the excitability of the left PPC by means of transcranial direct current stimulation (tDCS) modulates synchronization performance of the right hand. In order to determine the significance of the left PPC for superior synchronization in drummers, we here investigate the effects of cathodal and anodal tDCS in 20 professional drummers on auditory-motor synchronization of the right hand. A continuation and a reaction time task served as control conditions. Moreover, the interaction between baseline performance and tDCS polarity was estimated in precise as compared to less precise synchronizers according to median split. Previously published data from 16 non-musicians were re-analyzed accordingly in order to highlight possible differences of tDCS effects in drummers and non-musicians. TDCS was applied for 10 min with an intensity of 0.25 mA over the left PPC. Behavioral measures were determined prior to and immediately after tDCS. In drummers the overall analysis of synchronization performance revealed significantly larger tap-to-tone asynchronies following anodal tDCS with the tap preceding the tone replicating findings in non-musicians. No significant effects were found on control tasks. The analysis for participants with large as compared to small baseline asynchronies revealed that only in drummers with small asynchronies tDCS interfered with synchronization performance. The re-analysis of the data from non-musicians indicated the reversed pattern. The data support the hypothesis that the PPC is involved in auditory-motor synchronization and extend previous findings by showing that its functional significance varies with musical expertise.

## Introduction

Timing abilities are essential for precise movement execution, in particular when movements have to be executed with respect to external events. The ability to predict such events increases movement accuracy and reduces attentional demands. A well-established paradigm to investigate motor timing with respect to an external signal is the so-called synchronization task, which requires subjects to synchronize their own finger taps with respect to a regular metronome (reviewed in Repp and Su, 2013). Despite the simplicity of this task, non-musicians typically show the so-called negative asynchrony, which is characterized by the tap preceding the tone by several tens of milliseconds (for a review, see Repp and Su, 2013). Motor timing relies on a cerebello-thalamo-cortical network (Pollok et al., 2005; Pecenka et al., 2013; for reviews, see Coull and Nobre, 2008; Chen et al., 2009). Core timing functions have been related to the basal ganglia (Malapani et al., 1998) and the cerebellum, which has been particularly linked to the stabilization of movements with respect to external events (Ivry et al., 2002; Spencer et al., 2005; for a review, see Molinari et al., 2003) as well as to the anticipation of sensory events (Tesche and Karhu, 2000). On a cortical level precise motor timing engages parietal as well as primary motor and premotor areas (Karabanov et al., 2009; Pecenka et al., 2013; for a review see Coull and Nobre, 2008). A specific relevance of the dorsal premotor cortex (dPMC) for precise movement timing with respect to auditory stimuli has been found suggesting that the dPMC integrates auditory information with motor actions (Chen et al., 2006, 2008, 2009; for a review, see Zatorre et al., 2007).

Drummers (Krause et al., 2010a,b) and percussionists (Manning and Schutz, 2016) show superior synchronization performance as compared to non-musicians and even as compared to professional pianists (Krause et al., 2010a,b). This behavioral advantage has been related to a stronger functional interaction between the thalamus and the posterior parietal cortex (PPC), suggesting a specific significance of the PPC for synchronization accuracy (Krause et al., 2010b). The term synchronization accuracy refers to the mean temporal distance between the onsets of the auditory pacing signal and the finger-tap as well as its variability. Noteworthy, although the data by Krause et al. (2010b) reveal evidence for a stronger involvement of the dPMC in professional musicians as compared to non-musicians, the dPMC cannot account for superior synchronization in drummers, since no significant differences between drummers and pianists were found. The PPC has been related to sensorimotor integration possibly acting as sensorimotor interface (Andersen, 1997; Andersen and Buneo, 2003) as well as to anticipatory motor control, which suggests that movements can be planned and executed not only with respect to actual but also to anticipated sensory events (Beudel et al., 2009; reviewed in Blakemore and Sirigu, 2003). Due to the regularity of the pacing signal, the synchronization task allows the investigation of anticipatory motor control. In line with this hypothesis, faster reaction times with respect to temporally predictable visual cues have been particularly related to increased activation of the inferior PPC (Coull et al., 2016).

Although neuroimaging studies reveal important insights into brain areas involved in a certain task, the results do not necessarily allow a conclusion regarding their functional significance for task execution. In order to estimate the functional relevance of different brain areas within a network, non-invasive brain stimulation methods like transcranial direct current or magnetic stimulation (tDCS/TMS) can be applied. These methods allow the modulation of cortical excitability. Evidence exists that tDCS changes the resting membrane potential in a polarity specific manner. While anodal tDCS increases the likelihood of neural firing by depolarization of neurons, cathodal tDCS yields hyperpolarization of cell bodies (Lang et al., 2005; reviewed in Stagg and Nitsche, 2011; Shin et al., 2015). Stimulation after-effects are assumed to rely on changes of inhibitory and excitatory synapses (reviewed in Stagg and Nitsche, 2011).

Previous studies showed that modulation of the PPC excitability by 1 Hz repetitive TMS (rTMS; Krause et al., 2012) or tDCS (Krause et al., 2014) changes synchronization accuracy in non-musicians as indicated by larger tap-to-tone asynchronies following anodal tDCS (Krause et al., 2014) and smaller asynchronies following inhibitory 1 Hz rTMS (Krause et al., 2012). Since in non-musicians no significant effects were found in the continuation and reaction tasks, we hypothesized that the PPC is particularly involved in anticipatory motor control. The present study aims at investigating whether this area is causally involved in superior synchronization in professional drummers. To this end, anodal and cathodal tDCS was applied to the left PPC and effects on synchronization as well as continuation accuracy and reaction times of the right hand were determined. Assuming a specific significance of the PPC for anticipatory motor control, we expected effects of tDCS: (*i*) on synchronization accuracy only; and (*ii*) being evident particularly in precise drummers.

## Materials and Methods

### Participants

Twenty professional drummers (19 male) aged between 19 years and 63 years (34.3 ± 2.6 years; mean ± standard error of the mean; SEM) were included in the present study. Sample size was determined with respect to our previous study (Krause et al., 2014) revealing relatively large effect sizes. The mean lateralization ratio according to the Edinburgh Handedness Inventory (Oldfield, 1971) was 96.3 ± 0.7 indicating that all participants were right-handed. They were either students of a music college or worked as professional musicians in an orchestra or as music teachers. Nineteen participants were formally educated on the instrument for 13.7 ± 1.2 years on average (range 5–24 years). One participant learned the instrument by self-education without any formal training. Mean age at the beginning of formal training was 9.9 ± 1.3 years (range 3–21 years). Mean duration of regular practice was 17.2 ± 2.6 years.

In addition, data from 16 healthy non-musicians (6 male) with a mean age of 23.7 ± 1.0 years were re-analyzed (Krause et al., 2014) in order to determine a possible interaction between baseline performance and musical expertise on tDCS effects. Right-handedness was indicated by a mean lateralization ratio of 85.0 ± 4.3. This group was labeled non-musicians since they never had regularly practiced an instrument.

Subjects with personal or family history of epileptic seizures or other neurological or psychiatric disorders, cardiac pacemaker or intracranial metal implants or intake of central nervous system-effective medication were not included in the study.

### Ethics

The study was carried out in accordance with the standards set by the latest revision of the Declaration of Helsinki. Experimental procedures were approved by the local ethics committee (Heinrich-Heine University Duesseldorf; study number 3347). Participants gave their written informed consent prior to participation.

### Experimental Paradigm

Participants were naïve with respect to the exact hypotheses of the study. None of them had received electrical brain stimulation before. Participants and the main investigator were blinded with respect to the type of tDCS until the end of the experiment. To this end, a second investigator ran the DC stimulator which was covered by a paperboard in order to hide the exact stimulation type. The order of tDCS conditions was counterbalanced across subjects. Timing abilities were measured by means of the: (*i*) synchronization paradigm which was always followed by; a (*ii*) continuation task; and a (*iii*) simple reaction time task. The order of tasks (synchronization-continuation vs. reaction time tasks) was counterbalanced across participants and tDCS sessions. During synchronization the subjects were instructed to tap with their right index finger in synchrony with an auditory pacing signal presented with a regular stimulus onset asynchrony (SOA) of 900 ms. Length of the pacing signal was 100 ms. After 30 taps the pacing signal stopped and subjects continued with the same rhythm for another 30 taps (continuation task). Reaction times were measured with respect to the same auditory signal being presented at varying SOAs of 1.000, 1.500 and 2.000 ms. In total 60 reactions were recorded for each individual. Behavioral data prior to and immediately after tDCS were measured by a photoelectric barrier mounted on a pad. For stimulus presentation and recording of behavioral data E-Prime^®^ 2.0 software was applied (Psychology Software Tools, Sharpsburg, MD, USA). Prior to data acquisition a short practice run was implemented in order to familiarize the subjects with the tasks. No specific training was conducted. During each experimental session, subjects were comfortably seated in a reclining chair. They were instructed to relax and to keep their eyes open during the entire experiment.

### Transcranial Direct Current Stimulation

tDCS was applied using a battery driven DC stimulator (NeuroConn GmbH, Ilmenau, Germany) with a pair of rubber electrodes (3 × 3 cm^2^) nestling between saline-soaked sponges. According to our previous study (Krause et al., 2014) the stimulation electrode was fixed above the left PPC and the reference electrode was placed over the contralateral orbit (Nitsche and Paulus, 2000; Moliadze et al., 2010). Self-adhesive bandages (Coban^TM^, 3M Deutschland GmbH, Neuss, Germany) were used for the fixation of the electrodes. Anodal as well as cathodal tDCS was applied during rest for 10 min, respectively, with an intensity of 0.25 mA, resulting in a current density of 27.77 μA/cm^2^ below electrodes. In line with our previous study (Krause et al., 2014) the relatively weak stimulation intensity was chosen in order to adjust the current density for the electrode size. A previous study has shown that smaller stimulation electrodes in combination with lower intensities result in higher tDCS focality (Nitsche et al., 2007). We tried to reduce the probability of primary motor cortex (M1) co-stimulation by the application of 3 × 3 cm^2^ stimulation electrodes along with lower stimulation intensities. The current was ramped up and down over additional 10 s at the beginning and the end of stimulation, respectively. Impedance was kept below 10 kΩ. Mean impedance was 8.2 ± 0.4 kΩ. An interval of at least 1 week was interspersed between anodal and cathodal tDCS sessions in order to avoid carryover effects. Stimulation was in accordance with established safety protocols (Nitsche et al., 2003; Iyer et al., 2005). In order to monitor the quality of blinding, subjects were asked to estimate the respective stimulation condition by a questionnaire. To this end, at the end of each session they were asked to decide whether they had received either anodal or cathodal tDCS.

**Figure 1:**
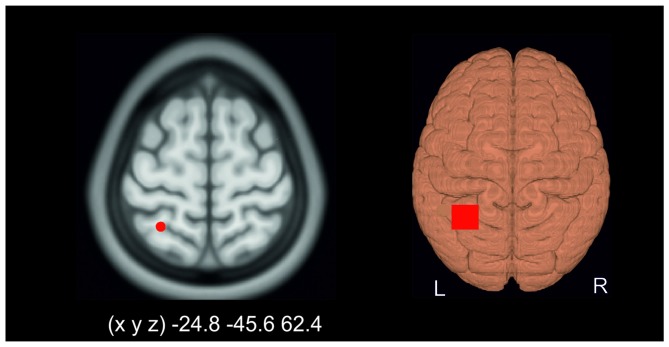
**Application of transcranial direct current stimulation (tDCS).** The target area was set to Talairach coordinates *(x, y, z)* −25, −46, 62 corresponding to Brodmann area (BA) 7 (left). The right part of the figure schematically illustrates the electrode montage over the left posterior parietal cortex (PPC). Please note that the reference electrode was placed over the contralateral orbit.

The PPC was localized by means of a neuronavigation system (LOCALITE, Sankt Augustin, Germany) using a standard brain. The stimulation target was set to the Talairach coordinates *(x, y, z)* −25, −46, 62 corresponding to Brodmann area (BA) 7 (Figure 1).

In order to ensure that the stimulated area does not overlap with the M1, M1 was localized by means of single pulse TMS using a standard figure of eight coil with an outer winding diameter of 80 mm (MC-B 70) being connected to a MagPro stimulator (Mag Venture, Hückelhoven, Germany). The coil was placed tangentially to the scalp with the handle pointing backwards and laterally at about 45° away from the midline inducing an initial posterior-anterior current flow in the brain. The magnetic stimulus had a biphasic waveform with a pulse width of about 300 μs. In a first step, the optimal cortical representation of the first dorsal interosseous (FDI) muscle was determined by eliciting motor evoked potentials (MEPs; for an overview see Kobayashi and Pascual-Leone, 2003). Then, the point which evoked the largest motor response of the FDI muscle was determined as motor hot spot by moving the coil in 0.5 cm steps anterior, posterior, medial and lateral to this area. The mean distance between the left M1 hot spot and the stimulated area corresponding to the left PPC was 4.6 ± 0.2 cm.

### Data Analysis

Synchronization performance and reaction times were determined as the temporal distance between tap and tone onsets. Continuation performance was determined as the mean inter-tap interval (ITI) and calculated by the temporal distance between two subsequent tap onsets. In addition, the inter-tap variability was calculated for the continuation task as determined by the mean standard deviation of the ITI. Accordingly, the tap-to-tone variability was determined for the synchronization task as indicated by the mean standard deviation of the temporal distance between tap and tone onsets. The first three taps of each run were excluded from the analysis. Data which were two standard deviations below or above individual and group means were identified as outliers and discarded. Less than 5% of individual data per condition were removed prior to the final analysis. The number of outliers did not significantly differ between stimulation conditions (*p* > 0.14). Due to this procedure synchronization and continuation data from one subject and reaction times from two other subjects were excluded. Analysis of variance (ANOVA) with factors *stimulation* (anodal vs. cathodal) and *time* (pre vs. post) were calculated for each task (synchronization, continuation, reaction), respectively. *T*-tests were applied for *post hoc* analysis.

In order to determine whether tDCS effects vary depending on baseline performance, the data were additionally split with respect to baseline median of the tap-to-tone asynchrony (synchronization task) and ITI (continuation task), respectively and were analyzed separately for subjects with performance levels above (large asynchronies) and below (small asynchronies) group median. A comparable procedure has been recently used for the investigation of tDCS effects on spatial attention depending on age (Learmonth et al., 2015). Median was calculated for each baseline measurement, respectively. In addition, data from our previous study investigating the effects of tDCS over the left PPC on motor timing in non-musicians (Krause et al., 2014) were re-analyzed with respect to baseline performance in the same way. This analysis aimed at investigating whether and to what extent effects of tDCS might vary with musical expertise and baseline performance.

## Results

### Blinding

In the first measurement 6 and in the second measurement 5 out of 20 participants correctly indicated the tDCS type, suggesting that blinding was successful.

### Synchronization Task

**Figure 2:**
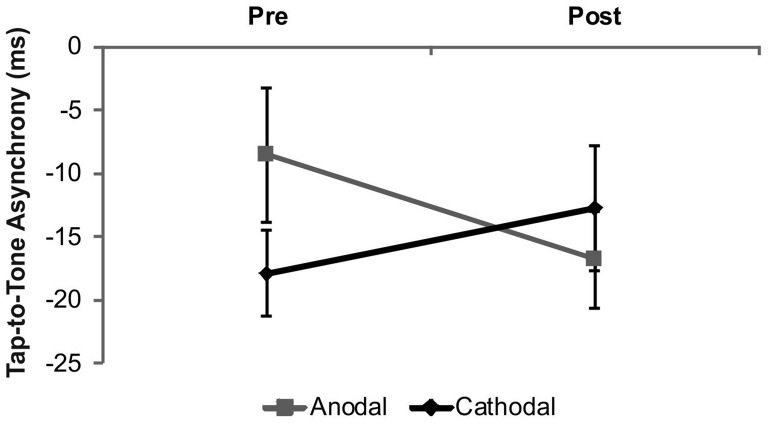
**Results from the overall analysis in drummers.** The analysis revealed a significant increase of the tap-to-tone asynchrony following anodal tDCS while no significant effects occurred following cathodal tDCS. Error bars indicate the standard error of the mean (SEM).

The analysis of the tap-to-tone asynchrony revealed a significant *stimulation* × *time* interaction (*F*_(1,18)_ = 6.71, *p* = 0.02; Figure 2). This interaction was due to larger tap-to-tone asynchronies following anodal stimulation as compared to baseline (*t*_(18)_ = 2.31, *p* = 0.03), while no significant effect following cathodal stimulation was found (*t*_(18)_ = −1.58, *p* = 0.13). Comparison of baseline performance between tDCS conditions revealed a trend towards significance (*t*_(18)_ = 1.97, *p* = 0.06). Neither a significant main effect of *stimulation* (*F*_(1,18)_ = 0.06, *p* = 0.45) nor *time* (*F*_(1,18)_ = 0.49, *p* = 0.49) was evident.

#### Effect of Age and Amount of Musical Training

Since the age at the beginning of formal musical training was quite variable across subjects, we compared subjects with early (i.e., starting the formal training below the age of 8 years) and late onset of musical practice (i.e., > 8 years) according to median split. The analysis did not reveal a significant main effect of *age* (*F*_(1,15)_ = 10.79; *p* = 0.23) or a significant interaction with this factor (*p* > 0.34). Moreover, no significant correlation: (*i*) between years of musical education; or (*ii*) duration of daily practice on the instrument with the amount of the tap-to-tone asynchrony was observed (*p* > 0.5).

#### Effects of Baseline Performance

**Figure 3:**
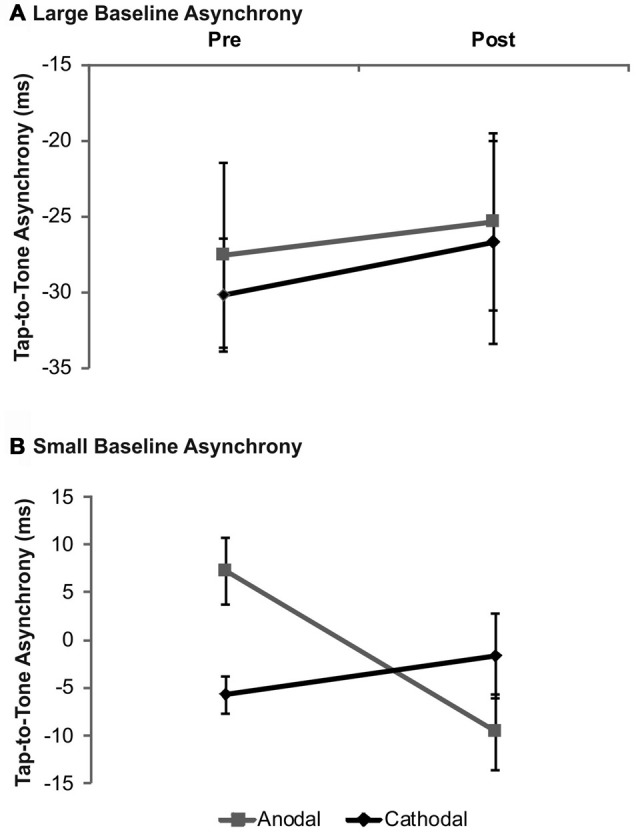
**Analysis of tDCS effects on synchronization accuracy depending on baseline performance in drummers.** Data were split according to group median of the tap-to-tone asynchrony during baseline. In drummers with large tap-to-tone asynchronies **(A)** no significant effects depending on tDCS polarity were observed while in participants with small asynchronies **(B)** anodal tDCS significantly modulated the tap-to-tone asynchrony. Error bars indicate the SEM.

Analysis for drummers with large baseline asynchronies according to median split revealed neither significant main effects (*stimulation*: *F*_(1,9)_ = 0.09, *p* = 0.78; *time*: *F*_(1,9)_ = 1.48, *p* = 0.26) nor a significant *stimulation* × *time* interaction (*F*_(1,9)_ = 0.83, *p* = 0.39). In drummers with small baseline asynchronies—however—the interaction turned out to be significant (*F*_(1,9)_ = 12.85, *p* = 0.01). *Post hoc* analyses revealed a significant shift from a mean positive to a mean negative asynchrony following anodal tDCS as compared to baseline (*t*_(8)_ = 3.94, *p* = 0.003) while no significant effect was found following cathodal tDCS (*t*_(8)_ = −1.09, *p* = 0.31). Mean asynchronies were significantly different during baseline (*t*_(8)_ = 4.14, *p* = 0.003), but not after tDCS (*t*_(8)_ = −1.24, *p* = 0.252). No significant main effect of factor *stimulation* was found (*F*_(1,9)_ = 0.22, *p* = 0.65) while factor *time* showed a trend towards significance (*F*_(1,9)_ = 4.82, *p* = 0.06). Data are summarized in Figure 3.

The observed baseline differences raised the question whether behavioral tDCS effects indeed occur due to tDCS or may arise simply due to such baseline differences. In order to estimate the effect of baseline performance on post-tDCS synchronization, we additionally calculated regression analyses with post-tDCS synchronization as the dependent and baseline performance as the predictor variable. The analysis revealed significant effects in drummers with large baseline asynchronies (anodal tDCS: *F*_(1,7)_ = 13.40, *p* = 0.001, *R*^2^ = 0.66, *β* = 0.81; cathodal tDCS: *F*_(1,7)_ = 6.13, *p* = 0.04, *R*^2^ = 0.47, *β* = 0.68). In drummers with small baseline asynchronies post-tDCS synchronization was not significantly associated with baseline performance in the anodal condition (*F*_(1,8)_ = 1.09, *p* = 0.328, *R*^2^ = 0.120, *β* = 0.346) while in the cathodal condition a trend emerged (*F*_(1,7)_ = 5.03, *p* = 0.060, *R*^2^ = 0.418, *β* = 0.647).

The analysis of synchronization variability as indicated by the standard deviation of the tap-to-tone asynchrony did not reveal significant main effects or interactions neither for the entire group nor for the sub-group analysis (*p* > 0.09).

### Continuation Task

The analysis of ITI and inter-tap variability across the entire group did not reveal significant main effects or an interaction (*p* > 0.13). In subjects with large ITIs, a significant main effect of *time* was found (*F*_(1,8)_ = 11.38, *p* = 0.01) which was due to smaller ITIs post tDCS (904.41 ± 4.09 ms) as compared to pre-tDCS ITIs (916.74 ± 2.95 ms). A trend emerged for *stimulation* (*F*_(1,8)_ = 3.57, *p* = 0.09) which was characterized by smaller ITIs in the cathodal (903.19 ± 4.09 ms) as compared to anodal tDCS (916.74 ± 5.23 ms). The *stimulation × time* interaction (*F*_(1,8)_ = 0.27, *p* = 0.62) was not significant. The analysis of the data from subjects with small ITIs revealed a trend for *time* (*F*_(1,8)_ = 4.90, *p* = 0.06) and a non-significant effect of *stimulation* (*F*_(1,8)_ = 0.009, *p* = 0.92). The *time × stimulation* interaction was again not significant (*F*_(1,8)_ = 0.30, *p* = 0.60). The trend of factor *time* can be explained by smaller ITIs prior to (882.91 ± 3.00 ms) as compared to post tDCS performance (897.18 ± 5.63 ms).

### Reaction Times

The analysis revealed neither significant main effects of factors *stimulation* (*F*_(1,17)_ = 0.08, *p* = 0.78) and *time* (*F*_(1,17)_ = 0.41, *p* = 0.53) nor a significant interaction (*F*_(1,17)_ = 1.29, *p* = 0.27).

### Synchronization in Non-Musicians

**Figure 4:**
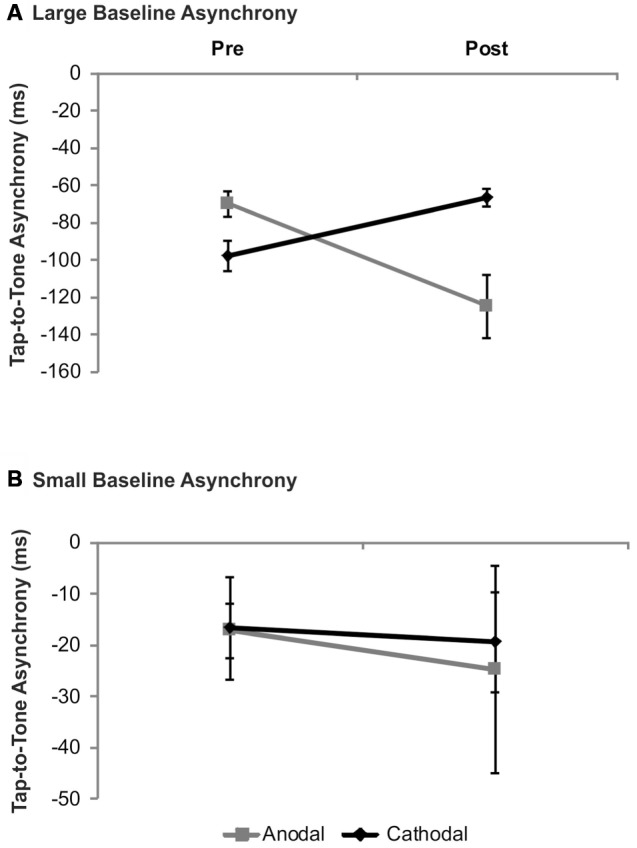
**Analysis of tDCS effects on synchronization accuracy depending on baseline performance in non-musicians.** Data were split according to group median of the tap-to-tone asynchrony during baseline. In non-musicians with large tap-to-tone asynchronies **(A)** anodal tDCS resulted in significantly larger asynchronies, while in subjects with small baseline asynchronies **(B)** no significant tDCS effects were found. Error bars indicate the SEM.

In order to test whether in non-musicians tDCS effects vary with baseline performance as shown in drummers, we re-analyzed the data published previously (Krause et al., 2014). In participants with small baseline asynchronies neither significant main effects of *stimulation* (*F*_(1,6)_ = 0.03, *p* = 0.86) and *time* (*F*_(1,7)_ = 0.28, *p* = 0.61) nor a significant *stimulation × time* interaction (*F*_(1,6)_ = 0.04, *p* = 0.85) emerged. In contrast to this, in participants with large baseline asynchronies a significant *stimulation × time* interaction (*F*_(1,6)_ = 14.21, *p* = 0.01) was evident suggesting larger tap-to-tone asynchronies following anodal tDCS (*t*_(6)_ = 2.80; *p* = 0.03) while following cathodal tDCS a trend towards smaller asynchronies was found (*t*(6) = −2.33; *p* = 0.06). Neither significant main effects of *stimulation* (*F*_(1,6)_ = 1.71, *p* = 0.24) nor *time* (*F*_(1,6)_ = 0.92, *p* = 0.37) were found. While the comparison of baseline asynchronies revealed a trend towards significance (*t*_(6)_ = 2.23, *p* = 0.07) significant differences emerged following tDCS (*t*(6) = −3.01, *p* = 0.024). Results are summarized in Figure 4.

Again, we calculated regression analyses with post-tDCS synchronization as dependent and baseline performance as predictor variable. In participants with small baseline asynchronies no significant effect in the anodal (*F*_(1,5)_ = 1.80, *p* = 0.24, *R*^2^ = 0.26, *β* = 0.51), but in the cathodal condition emerged (*F*_(1,5)_ = 9.55, *p* = 0.03, *R*^2^ = 0.66, *β* = 0.81). In participants with large baseline asynchronies no significant effect was found (anodal: *F*_(1,5)_ = 0.46, *p* = 0.53, *R*^2^ = 0.08, *β* = 0.29; cathodal: *F*_(1,5)_ = −1.64, *p* = 0.16, *R*^2^ = 0.35, *β* = −0.59).

Finally, we analyzed whether effects of tDCS on the size of the negative asynchrony is modulated by continuation performance. To this end analysis of covariance (ANCOVA) was calculated with pre-tDCS ITIs in the continuation task as co-variate. In drummers this analysis resulted in a non-significant effect of *time* in the anodal condition (*F*_(1,16)_ = 0.02, *p* = 0.89) while in non-musicians the significant effect of *time* remained unaffected (*F*_(1,11)_ = 5.11, *p* = 0.05). No modulation following cathodal tDCS was found for either group.

## Discussion

The analysis across the entire group suggests that anodal tDCS over the left PPC in professional drummers yields an increase of the tap-to-tone asynchrony of the right hand while no significant effect on reaction times was found. The data are in line with the hypothesis that the PPC is involved in precise auditory-motor synchronization, but not in motor control per se. The overall-effects in drummers resembled the effects observed in non-musicians (Krause et al., 2014). The sub-group analysis with respect to baseline performance suggests that tDCS influenced synchronization in drummers with small asynchronies only. In contrast to this, in non-musicians tDCS was found to modulate the tap-to-tone asynchrony in participants with large asynchronies. The data support the functional significance of the left PPC for auditory-motor synchronization of the right hand (Krause et al., 2012, 2014) and extend these findings by showing that behavioral tDCS effects vary depending on baseline performance and musical expertise. While in professional drummers the PPC seems to be relevant for exactly keeping the rhythm, in non-musicians, this area might be rather related to prevent the participants from deviating from a given pace within a broader range.

### Motor Timing in Musicians

Structural as well as functional reorganization in the musician’s brain is well-established (e.g., Schlaug, 2001; Münte et al., 2002; Gaser and Schlaug, 2003; Herholz and Zatorre, 2012). Early musical practice drives gray matter plasticity in the ventral premotor cortex (vPMC; Bailey et al., 2014) and white matter volume in the cerebellum (Baer et al., 2015). These changes were correlated with accuracy in an auditory rhythmic synchronization task, which requires subjects to synchronize their finger taps with respect to rhythms varying in their metrical complexity. The vPMC has been particularly related to visuo-motor integration (for a review, see Chen et al., 2009). Interestingly enough, activation changes in this area are not sensitive to the metrical structure of a rhythm as supported by a brain imaging study (reviewed in Chen et al., 2009), suggesting that it is less involved in higher-order aspects of movement control. In contrast to this, it has been suggested that the dPMC plays a crucial role for auditory-motor integration in a synchronization task (Pollok et al., 2008; for a review, see Chen et al., 2009). Findings by Chen et al. (2009) furthermore showed that activity within auditory cortices and the dPMC varies with metrical salience as determined by functional magnetic resonance imaging (fMRI).

A possible contribution of the dorsal PPC (BA 7) for motor timing in musicians has less attracted the literature so far. A recently published study suggests its involvement in the processing of rhythmic deviations in musicians after a short-term sensorimotor training (Lappe et al., 2016). This finding is in line with the hypothesis that processing of temporal and spatial stimuli relies on auditory as well as parietal and prefrontal brain areas (Di Pietro et al., 2004; Koch et al., 2009). The present results extend the current knowledge by providing evidence for the hypotheses that: (*i*) this area is indeed involved in anticipatory motor control; and (*ii*) that its functional involvement varies with the size of the baseline asynchrony as well as with musical expertise.

In contrast to the observed tDCS effects on synchronization performance, tDCS did not affect continuation performance as well as simple reaction times in a polarity-specific manner.

Nevertheless, the sub-group analysis of drummers with large baseline ITIs revealed a main effect of *time* as well as a trend towards significance of factor *stimulation*. The main effect of *time* was characterized by larger ITIs prior to as compared to post-tDCS performance. In contrast to this, in drummers with small ITIs a trend of this factor was due to larger ITIs post-tDCS as compared to pre-stimulation performance. Since we did not find a polarity-specific effect, we cannot exclude the possibility that this result reflects training or unspecific tDCS effects. Interestingly enough, results from the ANCOVA suggest that in drummers post-tDCS synchronization performance is modulated by pre-stimulation continuation efficiency. This finding suggests interdependency between both tasks in this group revealing evidence for the hypothesis that in drummers the PPC might be crucial for precise motor timing independent of a pacing signal. All in all this analysis may suggest that motor timing is differentially controlled in drummers as compared to non-musicians.

### PPC and Motor Timing

Precise motor timing is associated with a cerebello-thalamo-cortical network (Karabanov et al., 2009; Pecenka et al., 2013; for a review, see Coull and Nobre, 2008; Chen et al., 2009). Besides primary and premotor areas, the PPC has been suggested to be of particular importance for superior synchronization in professional drummers using magnetoencephalography (MEG; Krause et al., 2010b). Results from that study revealed evidence for a significantly stronger functional interaction between the thalamus and the PPC in professional drummers. Those data provide additional evidence for a stronger functional interaction between the thalamus and the dPMC, but this was found in professional pianists as well and thus this interaction less likely accounts for superior synchronization observed in drummers.

A recent study investigating patients with brain lesions following stroke suggests that lesions of the left but not the right PPC impair accuracy in the double-step task requiring the modification of an ongoing arm movement (Mutha et al., 2014). Accordingly, inhibitory TMS over the left PPC disturbed this ability (Desmurget et al., 1999) and increased parietal activation was shown when an ongoing action had to be modified (Mars et al., 2007). These data suggest a critical role of the left PPC for action modification in particular when movements were guided by actual and predicted sensory information. Furthermore, the present findings are in line with studies suggesting the involvement of parietal and premotor areas in motor timing (Coull et al., 2013; for a review, see Coull and Nobre, 2008). However, it should be stressed that those data reveal evidence for a specific relevance of the inferior PPC for precise timing while the present data reveal evidence for the contribution of its superior part. Given a stronger involvement of the inferior PPC in visuo-motor integration (for a review, see Chen et al., 2009), this apparent discrepancy can be explained by different modalities of sensory cues used in the studies: in the present study auditory pacing signals were applied while in the studies by Coull et al. (2013) visual stimuli were used.

The present data are in contrast with those from Vicario et al. (2013) showing overestimation of reproduced time intervals following cathodal tDCS over the right PPC and reduced variability following left PPC cathodal stimulation. It should be stressed that reproduction of temporal intervals in the supra-second range was investigated in that study, possibly requiring visuo-spatial attention. Thus, it is likely that the behavioral effects observed by Vicario et al. (2013) are mediated by attentional changes following right PPC tDCS. Combining those data with data from the present study, one may conclude that the PPC may differentially contribute to auditory-motor synchronization in the sub- and supra-second range. But, we realize that this interpretation remains speculative at the moment.

We argue in favor of a specific relevance of the PPC for auditory-motor synchronization, although tDCS may have affected somatosensory processing or auditory-somatosensory integration rather than motor control. Indeed previous studies suggest that tDCS applied to the PPC modulates multisensory integration of one’s own body (for a review, see Azañón and Haggard, 2009). Thus, it remains open whether the effect observed in the present study is indeed due to changes of auditory-motor synchronization or due to a modulation of the necessary somatosensory input.

### A Possible Contribution of the Primary Motor Cortex

Previous studies suggest that TMS (Koch et al., 2007; Karabanov et al., 2013) as well as tDCS over the PPC (Rivera-Urbina et al., 2015) yield changes of M1 excitability likely due to a modulation of functional connectivity between both areas (Rivera-Urbina et al., 2015). Since effects occurred at relatively long intervals of 10 and 15 ms, they are likely due to a modulation of polysynaptic pathways possibly involving the basal ganglia and/or the thalamus (Rivera-Urbina et al., 2015). The feasibility to affect cortical-subcortical connectivity by tDCS has been previously proven by combining neuroimaging studies with tDCS applied to M1 (Polania et al., 2012). Results from other studies reveal further evidence for monosynaptic connections between M1 and PPC (Koch et al., 2007; Koch and Rothwell, 2009; Karabanov et al., 2013), which were particularly found in the inferior parietal sulcus, an area that has been related to visuo-motor interaction (Cohen and Andersen, 2002; Grefkes and Fink, 2005). Regarding the dorsal PPC it was found that the strength of functional M1-PPC connectivity varies during learning being stronger at the beginning and returning back to baseline after training (Karabanov et al., 2012). Due to the simplicity of the synchronization paradigm and due to the fact that the subjects were not trained on the task, the effects observed here are less likely due to learning induced changes of M1 excitability. In addition, previous results from our group do not support this hypothesis since in non-musicians tDCS applied to M1 did not result in changes of the tap-to-tone asynchrony (Krause et al., 2014). Those data suggest that M1 seems to be involved more strongly in motor implementation rather than in motor timing. The assumption that M1 and PPC differentially contribute to motor control has been supported by a study investigating the effects of tDCS over both areas on skilled motor function (Convento et al., 2014). Finally, if the present results were indeed due to changes of M1 excitability, we would expect a general effect on movement execution independent of movement type. We would not exclude the possibility that PPC tDCS affected M1 excitability, but the observed behavioral effects appear to be less likely due to such changes.

### Why Does Anodal tDCS Increase the Asynchrony?

The primary mechanisms underlying tDCS effects are most likely alterations of the resting membrane state (for reviews, see Stagg and Nitsche, 2011; Shin et al., 2015). Based on stimulation effects on cortico-spinal excitability as determined by MEP changes, it has been suggested that anodal tDCS yields enhanced motor-cortical excitability while cathodal stimulation results in the reversed effect (for reviews, see Stagg and Nitsche, 2011; Shin et al., 2015). However, improved performance following cathodal tDCS has been found in attentional (Weiss and Lavidor, 2012) and complex perceptional tasks (Antal et al., 2004) as well as planning abilities (Dockery et al., 2009).

In a previous study we applied inhibitory rTMS over the left and right PPC, respectively and found smaller tap-to-tone asynchronies as compared to baseline following left PPC rTMS (Krause et al., 2012). Assuming that PPC is relevant for the comparison between predicted and actual sensory feedback (Blakemore and Sirigu, 2003), we brought forward the hypothesis that this potentially time-consuming mechanism is important for complex motor tasks, but detrimental to the relatively easy synchronization task (Krause et al., 2012).

We would like to stress that in precise drummers the asynchrony changed from a mean positive prior to tDCS to a mean negative asynchrony after stimulation, but the size of the asynchrony was comparable. Thus, for this group the assumption of larger tap-to-tone asynchronies following anodal tDCS does not sustain. Nevertheless, although the asynchrony was not found to be larger in terms of absolute values after anodal stimulation, the data suggest that tDCS interferes with synchronization accuracy, but the effect varies depending on baseline performance.

The present results support previous findings by Krause et al. (2014) and reveal further evidence for the involvement of the PPC in anticipatory motor control over motor control in general.

### Limitations

A main limitation of the present study is the lack of a sham condition. Thus, tDCS effects can be estimated by comparison with baseline performance only. Unexpectedly, the tap-to-tone asynchrony at baseline differed between tDCS conditions. This raises the question whether post-tDCS effects were driven by baseline differences. In order to clarify this issue, regression analyses were calculated. Assuming that post-tDCS synchronization performance was mainly driven by baseline differences, one would expect a significant regression in drummers with small and in non-musicians with large baseline asynchronies. However, this result was not provided by the analyses weakening the hypothesis that effects on synchronization accuracy occurred due to baseline differences. We would therefore argue that the modulation of synchronization performance indeed reflects a “real” tDCS effect, rather than an effect of pre-tDCS performance.

Another limitation of the analysis might be seen in the sub-group analysis with respect to the median split of baseline performance. This analysis: (*i*) does not consider the continuous nature of the outcome measures (i.e., synchronization and continuation); and (*ii*) does not allow the investigation of the most precise as compared to the most imprecise performance. Nevertheless, we would like to stress that the investigation of such extreme groups was not the aim of the present study. The data should be seen as a piece of evidence for the hypothesis that the functional significance of the PPC might vary depending on the interaction between baseline performance and musical expertise.

In addition, we acknowledge that drummers and non-musicians were not matched with respect to age and gender. For that reason we did not directly compare both groups. However, despite smaller tap-to-tone asynchronies in drummers, the overall effect of tDCS on synchronization performance was comparable in both groups. Thus, we would argue that the results of the sub-group analysis are less likely due to such group differences.

Finally, we realize that although the tap-to-tone asynchrony is usually negative in non-musicians, it can be even positive, particularly in musicians. Thus, the comparison of the tap-to-tone asynchrony between different conditions and groups might result in misleading findings (i.e., individuals always tapping close to the pacing signal will show a mean asynchrony comparable to other individuals producing larger but positive and negative asynchronies). Thus, mean values need to be interpreted cautiously, and a proper interpretation of synchronization data requires the consideration of the variability across subjects.

## Conclusion

The present results suggest that the functional relevance of the PPC for precise auditory-motor synchronization might differ depending on musical expertise. While in drummers the PPC might be relevant for keeping exactly the pace, in non-musicians the PPC might be rather related to prevent the participants from deviating from a given pace within a broader range.

## Author Contributions

BP: conception and design of the experiment, data collection and analyses, interpretation of the data, drafting the article; KS: data collection and analyses; AK: data collection, interpretation of the data, critical revision of the article; VK: conception and design of the experiment, data collection, interpretation of the data, critical revision of the article; NKS: conception and design of the experiment, interpretation of the data, critical revision of the article.

## Funding

BP is grateful for financial support by a grant from the Deutsche Forschungsgemeinschaft (DFG): PO806-3.

## Conflict of Interest Statement

The authors declare that the research was conducted in the absence of any commercial or financial relationships that could be construed as a potential conflict of interest.
